# The influence of demographic factors and health-related quality of life on treatment satisfaction in patients with gastroesophageal reflux disease treated with esomeprazole

**DOI:** 10.1186/1477-7525-3-4

**Published:** 2005-01-13

**Authors:** Alessio Degl' Innocenti, Gordon H Guyatt, Ingela Wiklund, Diane Heels-Ansdell, David Armstrong, Carlo A Fallone, Lisa Tanser, Sander Veldhuyzen van Zanten, Samer El-Dika, Naoki Chiba, Alan N Barkun, Peggy Austin, Holger J Schünemann

**Affiliations:** 1 AstraZeneca R&D, 431 83 Mölndal, Sweden; 2 Department of Clinical Epidemiology and Biostatistics, McMaster University, Hamilton, Ontario, Canada; 3 Department of Medicine, McMaster University, Hamilton, Ontario, Canada; 4 Division of Gastroenterology, McGill University Health Center, Montreal, Quebec, Canada; 5 AstraZeneca, Mississauga, Ontario, Canada; 6 Dalhousie University, Halifax, Nova Scotia, Canada; 7 Department of Medicine, School of Medicine and Biomedical Sciences, University at Buffalo, State University of New York, Buffalo, New York, USA; 8 Surrey GI Research/Clinic, Guelph, Ontario, Canada; 9 Department of Social & Preventive Medicine, School of Public Health and Health Professions, University at Buffalo, State University of New York, Buffalo, New York, USA

**Keywords:** Demography, esomeprazole, Feeling Thermometer, GERD, QOLRAD, treatment satisfaction

## Abstract

**Background:**

The correlation between treatment satisfaction and demographic characteristics, symptoms, or health-related quality of life (HRQL) in patients with gastroesophageal reflux disease (GERD) is unknown. The objective of this study was to assess correlates of treatment satisfaction in patients with GERD receiving a proton pump inhibitor, esomeprazole.

**Methods:**

Adult GERD patients (n = 217) completed demography, symptom, HRQL, and treatment satisfaction questionnaires at baseline and/or after treatment with esomeprazole 40 mg once daily for 4 weeks. We used multiple linear regressions with treatment satisfaction as the dependent variable and demographic characteristics, baseline symptoms, baseline HRQL, and change scores in HRQL as independent variables.

**Results:**

Among the demographic variables only Caucasian ethnicity was positively associated with treatment satisfaction. Greater vitality assessed by the Quality of Life in Reflux and Dyspepsia (QOLRAD) and worse heartburn assessed by a four-symptom scale at baseline, were associated with greater treatment satisfaction. The greater the improvement on the QOLRAD vitality (change score), the more likely the patient is to be satisfied with the treatment.

**Conclusions:**

Ethnicity, baseline vitality, baseline heartburn severity, and change in QOLRAD vitality correlate with treatment satisfaction in patients with GERD.

## Background

The inclusion of patients' opinions in the assessment of interventions has gained greater prominence over the last decades. Regulator agencies now call for the inclusion of patient-reported outcomes (PRO) in clinical trials evaluating pharmaceuticals interventions [1-4]. PRO of interest include health-related quality of life (HRQL), symptom assessment, and more recently, treatment satisfaction, in gastroesophageal reflux disease (GERD).

Whereas HRQL measures the patient's physical, psychological, and social level of function, treatment satisfaction assesses the patient's attitude towards the treatment, or the extent to which the patient is satisfied or not with the results of the treatment. Thus, treatment satisfaction focuses on the interaction of expectations and preferences for treatments and is defined as the individual's rating of important attributes of the process and outcomes of the treatment experience [5]. Coyne and co-workers [6] have summarized a number of patient important domains that describe satisfaction with treatment including symptom relief, flexibility with dosing, and treatment expectations. Treatment satisfaction is also associated with prescription regimens that involve less invasive dosing regimens [5,7-10], such as daily versus twice daily use [11].

Evaluating treatment satisfaction may assist healthcare providers in understanding the issues that influence adherence with therapeutic interventions. In addition, treatment satisfaction can be a useful PRO when treatments show similar efficacy because differences in satisfaction could lead to patient preferences for one treatment over another and greater adherence with various treatment regimens.

Demographic variables such as age, ethnicity, and gender may influence satisfaction [12]. Older people tend to be more satisfied with medical care than younger people [13-15], and Caucasian people on the whole are more satisfied than non-Caucasians [16]. In contrast, gender does not appear to influence treatment satisfaction [17].

The objectives of this study were to assess correlates of treatment satisfaction, including demographic factors, symptoms, and HRQL, as well as change scores in PRO instruments in patients with moderate to severe GERD receiving a proton pump inhibitor, esomeprazole.

## Methods

### Participants

No statistical determination of sample size has been done since the study is of exploratory nature. We enrolled 249 patients with GERD in 13 gastroenterology practices and four general practices across Canada between March 2002 and March 2003.

**Figure 1:**
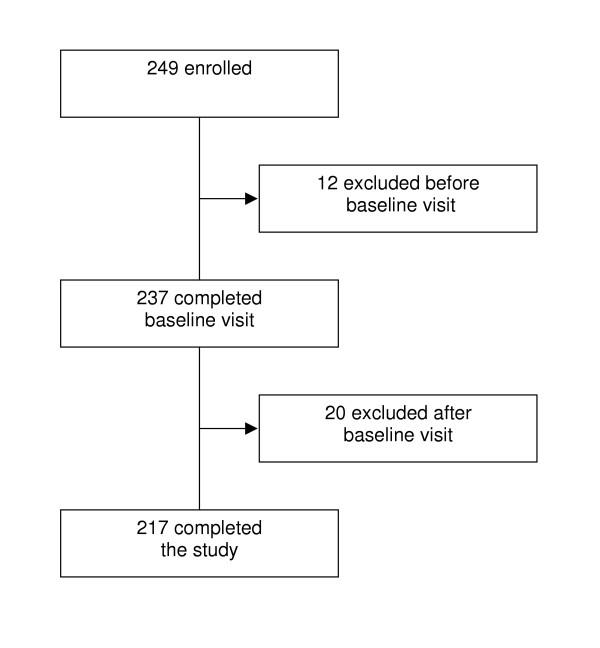
Flow chart

Included patients were 18 years of age or older and had a diagnosis of moderate to severe GERD and presence of symptoms for three months or longer [18]. Prior to inclusion all patients gave written informed consent in accordance with the Helsinki declaration. Of 249 patients, 217 (87%) completed the study. We excluded twelve patients because upon review they did not meet the initial inclusion criteria. Of the 20 patients who withdrew after the baseline visit, 4 withdrew because of adverse events, 2 were unwilling to continue, 4 were lost to follow-up and 10 were excluded because of improper administration or completion of the questionnaires at one visit. Figure 1 shows the flow of patients through the study. The final group of 217 completed patients received four weeks of therapy with esomeprazole 40 mg once daily, in the morning.

### Procedure

Patients completed PRO instruments at the clinic before and approximately 28 days after treatment. The completed PRO instruments included the Quality of Life in Reflux and Dyspepsia (QOLRAD) [19], the Feeling Thermometer (FT) [20], a four symptoms scale, the Standard Gamble (SG) [21], and an upper gastrointestinal (GI) symptom severity scale at baseline and follow-up. Patients completed the Health Utilities Index Mark 2 and 3 (HUI2 and HUI3) [22], and the Medical Outcomes Short-Form 36 (SF-36) [23] at baseline only; and the treatment satisfaction item at follow-up only. We describe these instruments below. In addition, trained research assistants collected information concerning demographic data and clinical data. Each visit lasted approximately 80 minutes.

### Treatment satisfaction

Patients rated their satisfaction with treatment on a seven point scale responding to the question: 'How satisfied are you with the study treatment you received?' with the response options: completely satisfied, very satisfied, quite satisfied, no change, dissatisfied, very dissatisfied, and completely dissatisfied.

### PRO instruments

#### QOLRAD

The QOLRAD is a 25-item disease-specific self-administered instrument asking about the impact of heartburn and acid regurgitation on the patient's HRQL during the previous week. The QOLRAD includes questions related to 5 domains; emotional distress, sleep disturbance, problems with food and drink, limitations in physical and social functioning, and lack of vitality. Patients respond to each question on a seven-point scale on which a higher score indicates better HRQL. The psychometric properties concerning validity, reliability, and responsiveness to change are reported elsewhere [19,24]. The minimal important difference (MID) that patients perceive as important is approximately 0.5 on the 1 – 7 scale [25].

#### FT

The FT is a visual analogue scale that resembles a thermometer. It is divided into 100 segments with a mark to represent each segment. Its anchors are dead (0) and full health (100) [21]. Patients mark their own health state and/or that of hypothetical patient scenarios or clinical marker states. In this study, three patient scenarios represented mild, moderate, and severe GERD. We developed and tested the clincal marker states with patients and clinicians [26]. The MID of the FT is approximately 6 on the 0 to 100 scale [27].

#### HUI

This is a 15 item questionnaire designed to quantify HRQL [22]. Each item has 4–6 response options. There are 8 attributes in the HUI3 classification system: vision, hearing, speech, ambulation, dexterity, emotion, cognition, and pain. In the HUI2 there are 7 attributes: sensation, mobility, emotion, cognition, self-care, pain, and fertility.

#### SF-36

The SF-36 contains 36 items that measure 8 dimensions: physical functioning, role limitations due to physical health problems, bodily pain, general health perceptions, vitality, social functioning, role limitations due to emotional problems, and general mental health. This questionnaire has been extensively tested for validation and reliability [23]. Each domain is scored on a 0 to 100 scale where higher scores indicate better HRQL. Scores on the SF-36 can also be expressed as two summary measures, the physical component score and the mental component score, which provide a measure of the overall effect of physical and mental impairment on HRQL.

#### Rating of four symptoms

To assess common symptoms in GERD, patients evaluated their heartburn, acid reflux, stomach pain, and belching for the past week using a seven-point scale ranging from no discomfort to very severe discomfort.

#### SG

The SG involves decision in the face of uncertainty, where in the standard administration the uncertainty involves a risk of death. The SG offers the patients two alternatives from which a choice must be made: Choice A is a hypothetical treatment with two possible outcomes: 1) returning to full health (probability p) for t years, at the end of which they die, or 2) immediate death (probability 1 – p). The alternative (choice B) is a certain outcome that he or she will stay in a health state (their own health state, or a patient scenario) for t years until death. t varies depending on the patient's age. The interviewer used a change board with the ping-pong approach varying the probability p in steps of 0.05 to find the value p where the respondent considered choice A = choice B. This value of p is the utility value for the health state in choice A in the interval from dead (0) to full health (1). The greater a patient's willingness to accept the risk of a worse outcome (e.g. dead) to avoid the health state in choice A, then the lower is the utility of the state in choice A to them.

#### Rating of upper GI symptom severity

Patients documented the severity of overall upper GI symptom on a seven-graded scale (1 = no symptoms; 7 = severe symptoms) over the past seven days. At baseline, patients who had no, minimal or mild symptoms were not included in this study.

### Statistical analyses

We calculated the mean and standard deviation of the basic demographic variables. Our multiple linear regression analysis focused on the outcome variable treatment satisfaction, which we treated as a continuous outcome variable. Evaluation of the data with polynomial regression yielded similar results. Potential correlates were demographic variables and baseline scores, as well as change scores for the PRO instruments described in the previous section. We first modelled these variables univariately as correlates of treatment satisfaction and only those that were significant at p < 0.1 entered into the multiple regression model. After having entered the multiple regression model, only those significant at p < 0.05 remained in the final model.

## Results

Table 1 shows the baseline demographic characteristics and frequencies of the included patients. The mean age was 50 years, and approximately 50% of the patients were female. The mean number of months since diagnosis was 86 months. Approximately 70% were full-time or part-time employed, and 88% were Caucasians.

**Table 1:** Demographic characteristics and frequencies at baseline for the study sample (N = 217).

	Frequency	Percentage
Gender		
Male	103	47.5
Female	114	52.5
Age		
Mean (SD)	49.7 (13.7)	
Range	20–82	
Months since diagnosis		
Mean (SD)	86.3 (99.4)	
Range	1–504	
Smoking history		
Never	94	43.5
Yes	38	17.6
Previous	84	38.9
Living alone	23	10.6
Employed: full-time and part-time	149	68.7
Ethnicity		
Caucasian	191	88.0
Other	26	12.0
Severity of gastroesophageal reflux disease (GERD)		
Moderate problem	112	51.6
Moderate severe problem	74	34.1
Severe problem	27	12.5
Very severe problem	4	1.8

**Figure 2:**
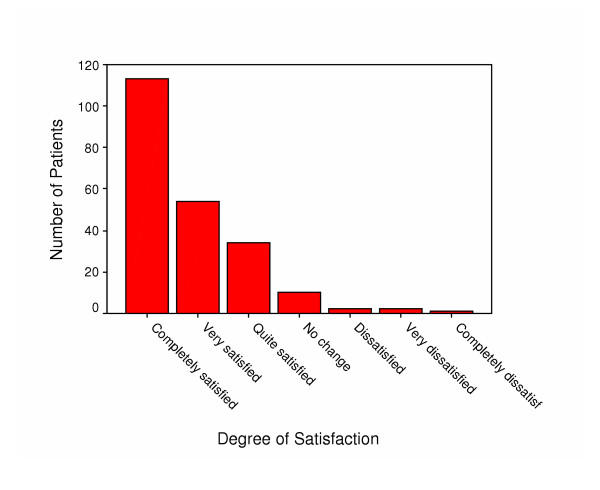
Distribution of treatment satisfaction scores

Table 2 depicts the mean baseline scores for the QOLRAD, the four symptoms scale, the FT, the SG, the HUI, and the SF-36. The mean QOLRAD scores at baseline were lowest for the food/drink domain, indicating worse HRQL for this domain, and the mean scores at baseline for the four symptoms show that patients had most problems with heartburn. Furthermore, the mean SF-36 scores at baseline were lowest (worse) for the bodily pain dimension, and highest (best) for the social functioning domain. Figure 2 shows the distribution of the treatment satisfaction scores. Approximately 50% of the patients were completely satisfied, 25% were very satisfied, and approximately 15% were quite satisfied. About 7% reported no change or dissatisfaction of different severity.

**Table 2:** Baseline scores for Quality of Life in Reflux and Dyspepsia (QOLRAD), four symptoms, Feeling Thermometer (FT), Standard Gamble (SG), Health Utilities Index Mark 2 and 3 (HUI), and Medical Outcomes Short Form-36 (SF-36).

	Mean	SD
**QOLRAD dimensions**		
Emotional distress	4.5	1.4
Sleep disturbance	4.5	1.5
Food/drink problem	3.8	1.2
Physical/social functioning	5.4	1.4
Vitality	4.3	1.3
**Four symptoms**		

Stomach pain	3.9	1.5
Heartburn	4.5	1.2
Belching	3.6	1.6
Acid reflux	4.1	1.6

**FT**	0.7	0.2
**SG**	0.8	0.2
**HUI2**	0.8	0.2
**HUI3**	0.8	0.2
**SF-36**		

Physical functioning	46.6	9.0
Role-physcial	45.5	11.4
Bodily pain	42.8	9.4
General health	46.2	9.7
Vitality	45.9	9.8
Social functioning	47.7	10.3
Role-emotional	46.5	12.0
Mental health	46.9	10.4
Physial component	45.1	8.7
Mental component	47.6	11.0

Table 3 portrays the results from the multiple linear regression analysis. Ethnicity, baseline QOLRAD vitality, baseline heartburn from the four symptoms scale, and QOLRAD vitality change score remained as independent variable when all variables had entered the model. Caucasian patients were more likely to be satisfied with the treatment than patients of other ethnicity. Higher baseline QOLRAD vitality scores, higher levels of heartburn and larger change on the QOLRAD vitality score were associated with greater treatment satisfaction.

**Table 3:** Results from the multiple linear regression analysis with treatment satisfaction as outcome variable.

Correlate variables	Parameter estimate (β)	SE	P-value
Ethnicity (Caucasian vs. other)	-0.570	0.190	0.003
QOLRAD Vitality baseline	-0.628	0.068	<0.001
Four symptoms Heartburn	-0.195	0.055	<0.001
QOLRAD Vitality change	-0.593	0.071	<0.001

Note. R^2 ^= 0.34 includes ethnicity, QOLRAD Vitality, heartburn, and QOLRAD Vitality change score

## Discussion

The objective of this study was to assess correlates of treatment satisfaction in patients with moderate to severe GERD receiving esomeprazole. We found that Caucasian ethnicity, greater vitality and more severe heartburn at baseline, correlates with treatment satisfaction. Furthermore, the greater the improvement on vitality change score, the more likely the patient is to be satisfied with the treatment.

The strengths of this study include the detailed assessment of a number of demographic characteristics, HRQL and symptoms. However, this study has two important limitations. First, we did not perform a placebo controlled trial limiting our ability to assess satisfaction as a true treatment result versus other reasons for satisfaction. Second, investigators have not conducted a thorough psychometric assessment of the treatment satisfaction instrument we used in this study.

Nevertheless, the present study yields four important results. First, in this sample of GERD patients without prior endoscopic evaluation of their symptoms, Caucasian ethnicity was positively associated with treatment satisfaction. Ethnic origin is perhaps one of the most complex demographic characteristics [12] and it has previously been reported that Caucasian people on the whole are more satisfied than non-Caucasians [16].

Second, higher vitality scores, as assessed by the QOLRAD, were associated with higher treatment satisfaction. A patient's health status prior to receiving treatment may cause the patient to be either more or less satisfied with treatment. Clearly and McNeil [28] reported positive correlations between health status and satisfaction. However, it is unclear if satisfaction was correlated with health status before intervention or with health status after intervention. A possible interpretation of the positive association between QOLRAD vitality and treatment satisfaction in our study might be that patients with a high vitality score at baseline are less distressed by their disease, and therefore tend to be more satisfied. The association in our study between higher vitality scores, as assessed by the QOLRAD, and higher treatment satisfaction is in line with Revicki and co-workers [29] who found that patients reporting greater severity in heartburn symptoms were more likely to report psychological distress and impaired well-being compared with those who reported no or mild symptoms. However, Revicki et al measured HRQL with a generic instrument while we used a disease-specific instrument.

Third, higher scores for heartburn, assessed with the four symptoms scale, were related to higher treatment satisfaction. Thus, in our study population, patients with high discomfort from heartburn at baseline perceived a high satisfaction with treatment.

Fourth, the higher the improvement on the QOLRAD vitality (change score), the more likely the person is to be satisfied with the treatment.

Patients' age is regarded as the most consistent determinant characteristic of satisfaction [13-15]. The results from this study did not reveal that treatment satisfaction was related to age. However, Fitzpatrick [30,31] and Fox and Storms [32] highlight the lack of consistency of the effect of age in satisfaction studies. Since satisfaction studies focused on a variety of concepts, such as satisfaction with medical care, satisfaction with hospital management, satisfaction with health services, and satisfaction with treatment, it might be that the association between age and satisfaction is dependent on the concept assessed. The lack of an association to age reveals also the possible that our study population was too homogenous with regard to age.

Although some studies have reported that patient gender affects satisfaction values [33,34], other studies did not find such association [17,35]. In line with this, in our study population treatment satisfaction was not associated with gender.

The current results may be unique to the study sample since no placebo control group was included in the study and, therefore, we were unable to evaluate whether the factors related to treatment satisfaction are related to real treatment effects or patients' need to please and placebo effects. The efficacy, tolerability and safety of esomeprazole versus other proton pump inhibitors has been shown in other studies [36-40]. In this study, patients had moderate to severe symptoms of GERD and some patients had received proton pump inhibitors prior to this study. The latter indicates that our study population is selected with regard to symptom severity, and mixed with regard to previous medication, which might limit generalizability of the findings. Treatment satisfaction in patients with mild GERD symptoms and with no previous experience of proton pump inhibitors remains unknown.

Investigators often use several PRO instruments, each with many dimensions and single items that are more or less correlated in clinical studies. This can lead to a large number of statistical tests being carried out and an increased risk of statistically significant findings occurring by chance in the absence of adjustment of P-values. In the present report we did not carry out adjustments for multiple comparisons for two main reasons. Firstly, the analysis of correlations was intended to be exploratory rather than confirmatory. Secondly, there is no consensus on how to adjust in analyses of the nature we conducted in this study. A simple adjustment according to Bonferroni would be too conservative, in part because many of the PRO variables are closely correlated.

Different drug therapies may elicit unwanted side-effects, which could compromise the patients' HRQL, and adherence with the treatment. Thus, a challenge in the management of GERD is to achieve as high adherence as possible. In addition, treatment satisfaction can be of use when different drug therapies show similar efficacy since it can lead to a preference for one drug over another and greater adherence.

Our study also supports the need for validated treatment satisfaction instruments because the available instruments vary widely in clinical trials [41] and the majority of studies rely on single items. There is a need for developing and improving psychometric documentation of instruments measuring treatment satisfaction [42].

## Conclusions

We examined correlates of treatment satisfaction, including demographic factors, symptoms, and HRQL, as well as change scores in HRQL, in patients with moderate to severe GERD who were not investigated by endoscopy. We observed that Caucasian ethnicity was positively related to treatment satisfaction. Furthermore, higher vitality and more severe heartburn were associated with treatment satisfaction. Finally, the higher the improvement on the QOLRAD vitality (change score), the more likely the patient is to be satisfied with the treatment.

## Authors' contributions

Alessio Degl' Innocenti was the project leader for this manuscript, edited the clinical protocol of the study, interpreted the data, and wrote the final manuscript as well as early versions. Gordon H Guyatt and Holger Schünemann were the principal investigators of the study, wrote the clinical protocol and grant application, are responsible for the study protocol, interpreted data and participated in writing the final as well as early versions of this manuscript. Ingela Wiklund contributed to the study protocol, interpreted the data and edited the manuscript. Diane Heels-Ansdell was responsible for the statistical analysis and edited the final manuscript as well as early versions. David Armstrong was co-principal investigator of the study, revised the clinical protocol, assessed patients, interpreted data and edited the final manuscript as well as early versions. Carlo A Fallone and Sander Veldhuyzen van Zanten revised the clinical protocol, assessed patients, interpreted data and edited the final manuscript as well as early versions. Samer El-Dika, Alan N Barkun, and Peggy Austin revised the clinical protocol, interpreted data and edited the final manuscript as well as early versions. Peggy Austin also co-ordinated the study. Lisa Tanser contributed to co-ordination of the study. All authors read and approved the final manuscript. The AstraZeneca global publications group approved the manuscript.
